# Progress in Surgery

**Published:** 1898-08-13

**Authors:** 


### Progress in Surgery.

### HERNIA.

**Cases of Special Interest.**—Mr. H. F. Waterhouse[1] records a case of double femoral hernia in a stout woman of forty-four, upon which he operated by a method given below. The hernia on the right side had a long thin neck, ending above in the crural canal, and below in a globular, tensely elastic swelling, obviously a cyst. The uppermost part of this swelling was a hernia, and the opinion was formed that by wearing a truss the lower part of the sac had become shut off by adhesions from the upper, and had subsequently become distended with fluid. The condition found at the operation, Mr. Waterhouse thought, was compatible with this theory. A septum shut off the space below from that containing the bowel. More than an ounce of serum was evacuated from the cyst.

Dr. Gore[2] recently operated upon a large scrotal hernia which had formed in a few days. A sudden strain of the groin in jumping a ditch seemed to have started the hernial protrusion. The hernia extended 15 inches downwards from the external abdominal ring, and reached to just above the internal condyle of the femur. Its circumference was 24 inches. At the operation, necessitated by vomiting and irreducibility, with a temperature of 102 deg., and pulse of 120, 15½ ounces of omentum were removed, and coils of small intestines were reduced; but it was found impossible to reduce the cæcum and appendix, which were also in the hernia, though it was of the left side. Reduction of the cæcum was only effected after extending the incision upwards and inwards into the abdomen. A good recovery ensued.

Mr. J. Hutchinson, jun.,[3] reports a case in which he operated upon a strangulated hernia which had been incompletely reduced by taxis. The rupture had developed suddenly, and was reduced by the practitioner on the second day; but the vomiting, anorexia, dryness of the tongue, abdominal pain, and slight fulness of the upper part of the right inguinal canal remained. On the third day Mr. Hutchinson operated by median laparotomy. A Richter's hernia was found tightly nipped in the internal abdominal ring. It retracted spontaneously after the ring had been incised upwards. Some of the peritoneum of the nipped portion of bowel had been stripped off by the tight strangulation. Uninterrupted recovery followed, but twelve months later laparotomy was again performed, for symptoms of mechanical obstruction. The small intestine was much distended, and some loops hanging in the pelvis appeared to be twisted. There was no band, but firm adhesion of a coil of ileum behind the right rectus muscle. No doubt this was the portion of bowel from which the peritoneum had been stripped twelve months before. No relief resulted, and in three days laparotomy was done a third time, and the adherent bowel dissected off the parietes. The patient died in a few hours. A post-mortem showed a volvulus of the small intestine which had passed through a small slit in the mesentery. The loop, twelve inches long, was twisted and gangrenous. Mr. Hutchinson remarks upon the difficulty of getting access to the internal abdominal ring by laparotomy, and upon the way in which symptoms vary when there are intestinal adhesions. A patient with wholesale adhesions may enjoy good health, whereas a single adhesion may lead to fatal results.

A case of strangulated diaphragmatic hernia, with rupture of the colon into the thorax, has recently been described by Bérard and Gallois.[4] The patient, a woman of twenty-five, had the cæcum opened after twelve days' intestinal obstruction and three days' vomiting. There was great relief for two days. Then she became suddenly collapsed, with pain in the left side of the thorax. A pneumo-thorax formed there, and fœtid fluid collected. The cavity was drained by incision. The patient died in thirteen days later with pneumonia. A post-mortem showed that the descending colon and sigmoid flexure had passed into the thorax through an aperture in the diaphragm. The bowel had ruptured close to this opening, to which it was adherent. The authors regard the hernia as an acquired one, and thought that it had formed directly after labour 18 months previously, for there was a history of a violent attack of pain in the left side at that period, lasting four days. Mr. J. W. Smith[4] remarks, in commenting on this case, that the diagnosis of diaphragmatic hernia has very rarely been made during life. Even laparotomy and careful search has failed to discover this lesion. In traumatic cases the rent can be sutured by a transpleural operation. Paolo, in 1890, reported seven successful cases, three being his own.

[1] Practitioner, Vol. I., 1898. [2] Brit. Med. Jour., May, 1898. [3] Lancet, Mar., 1893. [4] Med. Chron., April, 1898.

